# Targeting the chemokine-Treg axes in tumor immune evasion: from mechanisms to therapeutic opportunities

**DOI:** 10.3389/fimmu.2026.1779388

**Published:** 2026-03-11

**Authors:** Chao Lian, Ling Liu, Xuanfen Zhang

**Affiliations:** 1 Department of Plastic Surgery, The Second Hospital & Clinical Medical School, Lanzhou University, Lanzhou, Gansu, China; 2 Department of Plastic and Aesthetic Surgery, Changzhi People’s Hospital, The Affiliated Hospital of Changzhi Medical College, Changzhi, Shanxi, China; 3 Department of Reproductive Medicine, The Second Hospital & Clinical Medical School, Lanzhou University, Lanzhou, Gansu, China

**Keywords:** cancer immunotherapy, chemokine receptors, chemokines, immune evasion, regulatory T cells, tumor microenvironment

## Abstract

Cancer immunotherapy has transformed oncology, yet its clinical efficacy is often limited by immune evasion within the tumor microenvironment (TME). Regulatory T cells (Tregs), a key immunosuppressive lineage, potently inhibit effector T-cell proliferation and activation, thereby dampening antitumor immune responses. Tregs are frequently enriched in diverse solid tumors, and their abundance correlates with poor prognosis, increased tumor invasiveness, and therapeutic resistance. A major mechanism driving this enrichment is the chemokine-chemokine receptor axis. Tumor cells, along with other stromal and immune cells in the TME, secrete chemokines including CCL22, CCL20, and CXCL12, which bind to CCR4, CCR6, and CXCR4 on Tregs and direct their recruitment and activation within the TME. This establishes an immunosuppressive niche that promotes tumor growth, facilitates metastasis, and reduces responsiveness to immunotherapy. This review consolidates eight experimentally validated chemokine-Treg axes from 2005 to 2025, with each study annotated by tumor type and represented by the highest observed level of evidence. A systematic representation illustrates how these axes mediate Treg-driven immunosuppression and maps their prevalence across cancers. Focusing on these axes provides mechanistic insights, highlights potential therapeutic targets, and identifies predictive biomarkers. Strategies targeting the chemokine-chemokine receptor axes, including selective receptor blockade, combination with immune checkpoint inhibitors, and omics-based approaches to resolve Treg heterogeneity, offer avenues to reprogram the immunosuppressive TME and enhance antitumor immunity.

## 1 Introduction

The advent of cancer immunotherapy marks a transformative milestone in oncology. Unlike traditional therapies that directly target tumor cells, immunotherapy harnesses the immune system to eliminate malignancies. By coordinating the activation of T cells and other immune effectors, it aims to mount a robust antitumor response (1). Clinical outcomes of cancer patients critically depend on the ability of immune cells to recognize and eradicate malignant cells (2). Numerous studies have demonstrated a strong correlation between immune cell infiltration in the tumor microenvironment and patient prognosis across various solid tumors (3–5). In this context, regulatory T cells (Tregs) have emerged as a pivotal subset of immunosuppressive lymphocytes and have been recognized for their central role in modulating tumor immunity.

Tregs are a key subset of immunosuppressive lymphocytes that play a central role in tumor immune evasion (6–8). In 2004, Curiel et al. reported in Nature Medicine that, in ovarian cancer, tumor cells and M2-polarized macrophages recruit Tregs into the tumor microenvironment, where they suppress tumor-specific T-cell activity and thereby facilitate tumor growth and metastasis (9). Importantly, the study revealed a negative correlation between Treg density and patient survival. Subsequent studies have corroborated these findings across multiple solid tumors, including hepatocellular carcinoma, lung cancer, pancreatic cancer, gastric cancer, breast cancer, and melanoma, consistently showing that higher Treg infiltration is associated with increased tumor invasiveness, metastatic potential, and poorer prognosis (10–15). Mechanistically, Treg accumulation is closely linked to chemokine-chemokine receptor interactions, in which tumor- and stromal-derived chemokines direct Treg trafficking into the tumor microenvironment (16). These observations underscore the need to elucidate the molecular signaling networks governing chemokine-mediated Treg recruitment and their contribution to tumor immune evasion, providing potential targets for therapeutic intervention.

## 2 Cellular orchestrators of tumor immune suppression

The immunosuppressive tumor microenvironment (TME) arises from coordinated interactions among tumor cells, stromal components, and infiltrating immune cells. Dysregulated production of cytokines and chemokines plays a pivotal role in regulating immune cell recruitment, persistence, and functional polarization. Rather than acting independently, these soluble mediators form interconnected signaling networks that promote immune tolerance and facilitate tumor progression across cancer types (17, 18).

Chemokines are key determinants of TME composition, directing the trafficking of immunosuppressive immune subsets. Multiple chemokine-receptor axes drive the accumulation of Tregs, myeloid-derived suppressor cells (MDSCs), and tumor-associated macrophages (TAMs). CCR4 ligands, including CCL17 and CCL22, together with CCR6-CCL20 signaling, preferentially recruit and retain Tregs within tumors (8, 16). Similarly, CCR5 ligands (CCL3, CCL4, CCL5) and CCR2 ligands (CCL2) mediate the recruitment of MDSCs and monocyte precursors, which differentiate into suppressive myeloid populations (19, 20). These overlapping chemokine gradients establish a TME enriched in regulatory lymphoid and myeloid cells (17, 18). Following recruitment, cytokines such as GM-CSF, IL-10, and TGF-β support the survival, expansion, and functional polarization of these suppressive immune cells. IL-10 and TGF-β stabilize Treg suppressive programs, drive M2-like polarization of TAMs, and inhibit effector T-cell activity, thereby converting immune infiltration into durable immunosuppression (17–19).

Tregs, MDSCs, and TAMs serve as principal cellular mediators of tumor immune evasion. Tregs suppress CD8^+^ T cells and natural killer (NK) cells via FoxP3-dependent mechanisms, including CTLA-4-mediated inhibition of antigen-presenting cells and secretion of IL-10 and TGF-β (8, 16). MDSCs inhibit effector lymphocyte proliferation and activation through arginase-1, nitric oxide, and reactive oxygen species (ROS), while reinforcing Treg accumulation (19, 20). TAMs further consolidate immune suppression by producing IL-10, TGF-β, and immune checkpoint ligands, and by engaging in chemokine-mediated crosstalk with tumor cells (21–23). Microenvironmental stressors such as hypoxia, metabolic competition, and adenosine accumulation further amplify immunosuppressive signaling. These factors enhance PD-1/PD-L1 and CTLA-4 checkpoint activation, ultimately contributing to T-cell dysfunction and exhaustion (17, 18).

Collectively, cytokine-chemokine networks act as upstream orchestrators of tumor immune suppression by coordinating immune cell trafficking, polarization, and functional inhibition. The integrated actions of Tregs, MDSCs, and TAMs establish a stable immunosuppressive ecosystem, enabling tumor immune escape and limiting the efficacy of anticancer immunotherapies.

## 3 Regulatory T cells in tumor immune evasion​

### 3.1 Fundamental biology of Tregs

Tregs are a specialized subset of CD4^+^ T lymphocytes that play essential roles in maintaining immune homeostasis, restraining excessive inflammation, and preventing autoimmunity (24). In the context of cancer, however, these physiological functions are frequently co-opted to facilitate tumor immune evasion (25, 26). Tregs are broadly classified into thymus-derived natural Tregs (nTregs) and peripherally induced Tregs (iTregs), the latter arising from naïve CD4^+^CD25^-^ T cells under specific cytokine milieus. Notably, substantial phenotypic and functional overlap between these subsets complicates their discrimination within peripheral tissues and tumors (27).

Under physiological conditions, Tregs comprise approximately 5-10% of circulating CD4^+^ T cells. Their lineage identity and suppressive function are critically dependent on the transcription factor FoxP3, which orchestrates Treg development, stability, and functional specialization (28). While indispensable for immune tolerance, Tregs exhibit a context-dependent duality in cancer. On the one hand, they limit tissue-damaging inflammation; on the other, their accumulation within tumors suppresses antitumor immune responses, thereby promoting immune escape and disease progression (29).

### 3.2 Immunosuppressive functions of Tregs in the tumor microenvironment

TME constitutes a complex and dynamic ecosystem composed of malignant cells, immune infiltrates, stromal fibroblasts, endothelial cells, extracellular matrix (ECM), and a spectrum of soluble mediators (30). The reciprocal crosstalk among these cellular and molecular components orchestrates tumor initiation, progression, metastasis, and therapeutic responsiveness (31). Within this immunologically active milieu, Tregs represent a dominant suppressive subset that enforces peripheral tolerance and profoundly shapes the balance between tumor immunity and immune evasion. Tregs mediate multifaceted immunosuppressive functions through both cytokine-dependent and contact-dependent mechanisms. They produce immunoregulatory cytokines, such as TGF−β and IL−10, which modulate effector T-cell responses and influence antigen-presenting cell function (32, 33). Through engagement of CTLA−4, Tregs downregulate CD80/CD86 on antigen-presenting cells, attenuating costimulatory signaling and T cell priming (34). Moreover, Tregs promote the accumulation of immunosuppressive metabolites, including adenosine, via the CD39/CD73 ectonucleotidase axis, further dampening local immune activation (35, 36). Collectively, these mechanisms enable Tregs to establish and maintain an immunosuppressive tumor microenvironment, thereby facilitating tumor immune escape, progression, and resistance to immune checkpoint blockade.

### 3.3 Mechanisms of Treg enrichment in the tumor microenvironment

#### 3.3.1 Chemokine-mediated recruitment of Tregs

Chemokine-receptor interactions are a major mechanism by which Tregs are recruited into TME (37). Tumor cells, together with stromal and immune cells such as macrophages and NK cells, release chemokines that bind to their corresponding receptors on Tregs, guiding their migration and promoting local immunosuppression (38). This process has been observed across multiple tumor types. In breast cancer, inflammatory signals within the tumor microenvironment induce CCL22 production by tumor cells, which recruits CCR4^+^ regulatory T cells and promotes local immunosuppression (39). In gastric cancer, CCL20 produced by tumor cells facilitates Treg infiltration by CCR6 (40). Similarly, in EBV-associated nasopharyngeal carcinoma, upregulation of CXCL12 by tumor cells drives Treg chemotaxis through CXCR4, contributing to immune evasion and metastatic spread (41). Together, these findings illustrate that chemokine-mediated trafficking is a conserved and central pathway for Treg enrichment within tumors, underscoring its significance for tumor immune regulation and its potential as a therapeutic target.

#### 3.3.2 Local expansion and in situ conversion

Within the tumor microenvironment, Tregs are enriched not only through recruitment but also through local expansion and the conversion of naïve CD4^+^ T cells into iTregs (42). Soluble mediators such as TGF−β, IL−10, and low concentrations of IL−2 create a microenvironment that both promotes the proliferation of existing Tregs and drives the differentiation of naïve T cells into functional iTregs (43). For example, pancreatic cancer-derived TGF−β effectively converts peripheral or tissue-resident naïve CD4^+^ T cells into Tregs, an effect further enhanced by low IL−2 levels (44). M2-polarized macrophages contribute by establishing a cytokine milieu that supports both iTreg generation and Treg expansion (45). Although Tregs are intrinsically less proliferative than effector T cells, exposure to tumor-derived TGF−β and IL−10 stimulates their proliferation, enlarging the immunosuppressive compartment and reinforcing tumor-mediated immune evasion (8, 16). Together, these processes highlight the combined contribution of local expansion and *in situ* conversion in sustaining a high-density Treg population within tumors, strengthening immunosuppression and facilitating immune escape.

#### 3.3.3 Enhanced survival through apoptosis resistance

Tregs within TME exhibit increased survival compared with conventional effector T cells, which contributes to their sustained immunosuppressive function. Upregulation of anti-apoptotic members of the BCL-2 family within tumor-infiltrating Tregs, such as BCL-XL, promotes their survival by protecting against programmed cell death, and pharmacological targeting of these proteins can selectively induce apoptosis of tumor-infiltrating Tregs and enhance antitumor immunity (46). Metabolic adaptations and local microenvironmental stressors, including hypoxia and nutrient deprivation, further shape Treg survival pathways, enabling Tregs to persist despite hostile conditions and maintain high density within tumors (47). Signaling through co-stimulatory or checkpoint molecules such as GITR may modulate Treg stability and function, influencing their maintenance in the tumor microenvironment; however, direct evidence linking such signaling to resistance specifically against T cell receptor-induced apoptosis remains limited (48). This enhanced survival allows Tregs to continue suppressing cytotoxic lymphocyte activity and facilitate tumor immune evasion, highlighting apoptosis resistance and survival adaptation as important contributors to sustaining the immunosuppressive niche.

## 4 Chemokines and their receptors

Chemokines are a family of chemotactic cytokines comprising approximately 50 ligands. These ligands are classified into four subfamilies—XCL, CCL, CXCL, and CX3CL—based on the positions of their first two N-terminal cysteine residues. Chemokines signal through more than 20 G protein-coupled receptors and often function as homo- or heterodimers to modulate signaling. Correspondingly, chemokine receptors are categorized into four classes: XCR, CCR, CXCR, and CX3CR, reflecting their structural similarity to the ligands. A chemokine receptor can bind one or multiple chemokines. For example, CCR6 binds only CCL20, whereas CCR4 binds both CCL17 and CCL22. Most receptors are promiscuous, but a few are highly specific, binding only to a single chemokine (49–51).

### 4.1 Function and role of the chemokine-chemokine receptor axes in cancer

Chemokines and their receptors constitute a key signaling system involved in tumor development and progression. Through regulating immune cell recruitment and modulating interactions between tumor cells and the immune microenvironment, chemokine-chemokine receptor axes influence tumor immune status in a context-dependent manner, contributing to either immune suppression or antitumor immunity (52).

Increasing evidence suggests that distinct tumor types preferentially engage specific chemokine-chemokine receptor axes to establish immunosuppressive microenvironments. In gastric cancer, elevated expression of CCL20 in tumor epithelial cells has been reported, leading to activation of the CCL20-CCR6 axis. This pathway promotes the recruitment of CCR6^+^ immunosuppressive cell populations, including regulatory T cells, tumor-associated macrophages, and myeloid-derived suppressor cells. The accumulation of these cells is associated with reduced infiltration and impaired function of CD8^+^ T cells, consistent with a role in immune escape and tumor progression (40).

Notably, the cellular origin of CCL20 and its immunological consequences appear to be tumor-type specific. In hepatocellular carcinoma, CCL20 is predominantly produced by tumor-associated macrophages rather than tumor cells. Tumor-derived signals can enhance CCL20 secretion from macrophages, thereby reinforcing the CCL20-CCR6 axis and promoting the recruitment of CCR6^+^ regulatory T cells (53). This macrophage-Treg-associated chemokine circuit has been linked to diminished cytotoxic T-cell activity and reduced responsiveness to immune checkpoint blockade, suggesting a mechanism of immune evasion mediated by myeloid-lymphoid crosstalk.

In contrast, pancreatic tumorigenesis is characterized by engagement of a different chemokine axis. During the progression from pancreatic intraepithelial neoplasia to pancreatic ductal adenocarcinoma, pancreatic epithelial cells secrete CCL2, which activates the CCL2-CCR2 axis. This signaling pathway preferentially recruits immunosuppressive macrophages and myeloid-derived suppressor cells, contributing to the formation of a myeloid-dominant suppressive microenvironment, with relatively limited involvement of regulatory T cells (54). These observations indicate that chemokine-mediated immune suppression is shaped by tumor-specific programs rather than a uniform mechanism.

Importantly, chemokine signaling does not exclusively promote immune suppression. Certain chemokines, such as CXCL9 and CXCL10, have been associated with the recruitment of natural killer cells and CD8^+^T cells, supporting antitumor immune responses. The net immune outcome within the tumor microenvironment therefore reflects the balance between opposing chemokine-driven programs (55).

Beyond immune regulation, chemokine-chemokine receptor signaling directly shapes tumor cell behavior via pathways such as JAK/STAT, MAPK/ERK, and PI3K/AKT, regulating proliferation, survival, stress adaptation, and cytokine-chemokine production, thereby reinforcing feedback control within the tumor microenvironment (56).

### 4.2 Chemokine-Treg axes across tumor types in tumor immune evasion

**Figure 1:**
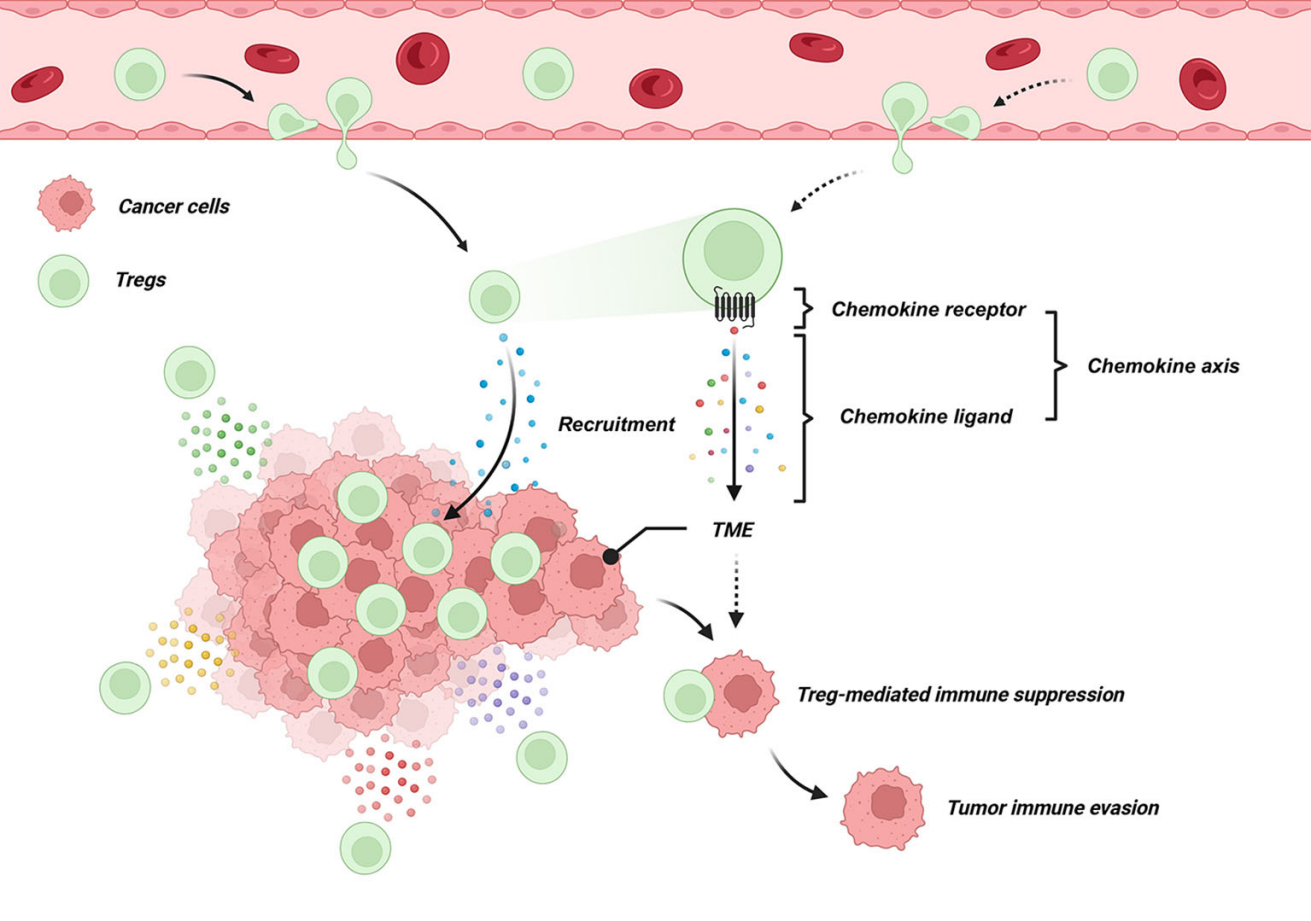
Chemokine-chemokine receptor axes mediate Treg recruitment and tumor immune evasion in the TME. Chemokine axes consist of paired chemokines and their cognate receptors. Tumor cells secrete chemokines that bind to chemokine receptors on Tregs, triggering chemotactic migration into the TME. The accumulation of Tregs promotes an immunosuppressive TME and supports tumor immune evasion.

Chemokines are fundamental regulators of immune cell trafficking, with lymphocyte migration determined by the spatial and temporal expression patterns of chemokines and their cognate receptors (57). Recruitment of Tregs follows this same principle and is mediated through defined chemokine-chemokine receptor interactions. The frequent enrichment of Tregs within tumor tissues reflects chemokine-driven homing and retention in the tumor microenvironment, where Tregs suppress antitumor immune responses and thereby promote immune evasion and tumor progression. The general mechanisms underlying chemokine-guided Treg recruitment and function in tumors are schematically illustrated in **Figure 1**.

This review focuses on tumor-derived chemokines and their cognate receptors on regulatory T cells that mediate tumor immune evasion within the tumor microenvironment. Literature published between 2005 and 2025 was reviewed to identify experimentally validated chemokine-Treg axes. Studies were included if they provided direct experimental evidence supporting a role for tumor-derived chemokines in Treg recruitment or function, and excluded if chemokines were primarily derived from stromal or immune cells or if functional validation was lacking.

**Figure 2:**
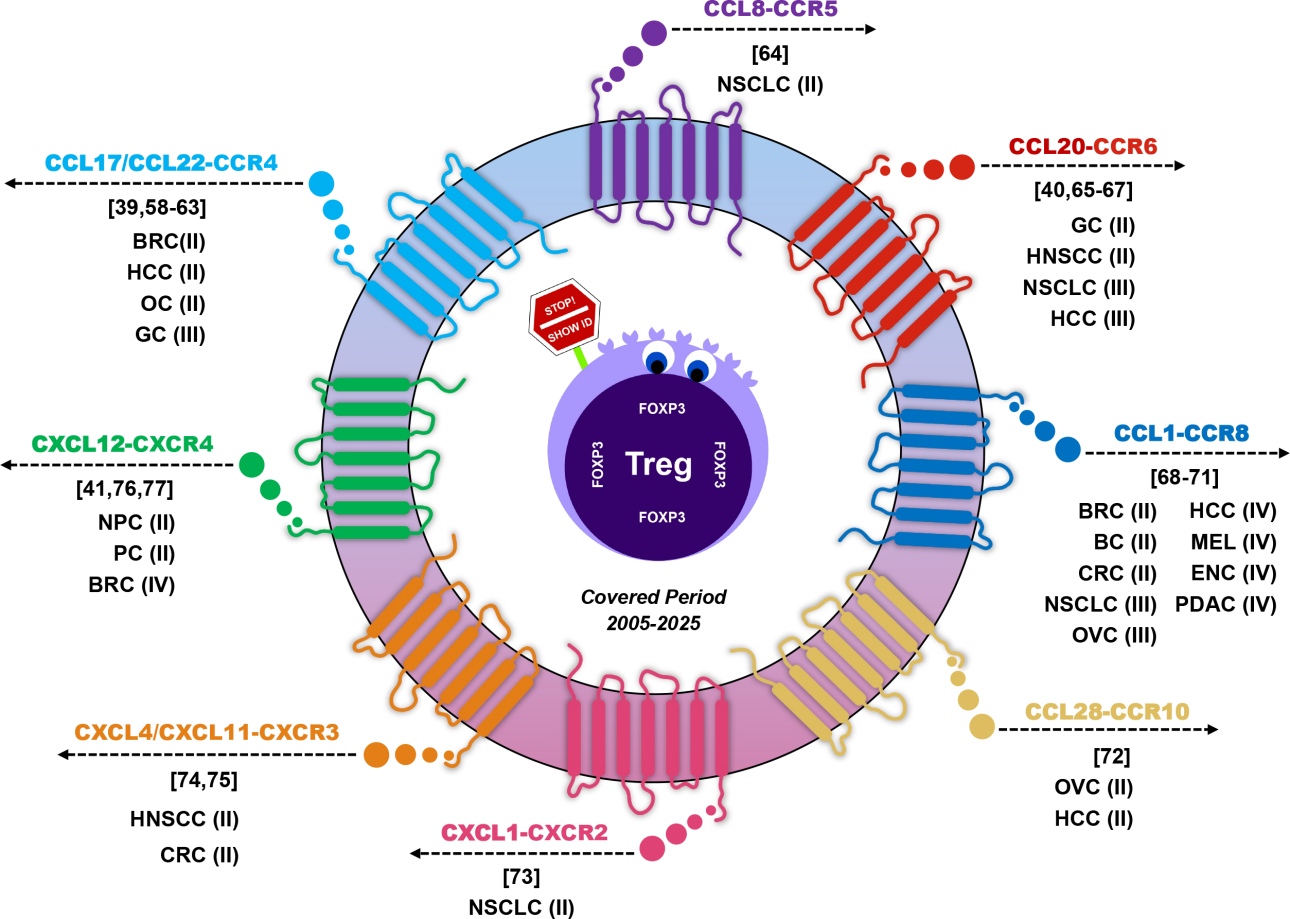
Chemokine-Treg axes in tumor immune evasion across cancer types. Eight chemokine-Treg axes reported in the literature are shown, with each axis annotated by tumor type, reference number, standardized abbreviations, and the level of experimental validation, providing a comparative overview of evidence strength. Abbreviations: BRC, Breast Cancer; HCC, Hepatocellular Carcinoma; OC, Oral Cancer; GC, Gastric Cancer; NSCLC, Non-Small Cell Lung Cancer; HNSCC, Head and Neck Squamous Cell Carcinoma; BC, Bladder Cancer; CRC, Colorectal Cancer; OVC, Ovarian Cancer; MEL, Melanoma; ENC, Endometrial Cancer; PDAC, Pancreatic Ductal Adenocarcinoma; NPC, Nasopharyngeal Cancer; PC, Prostate Cancer.

To reflect the strength of experimental support, each study was categorized according to its highest level of evidence: (I) clinical studies; (II) *in vivo* preclinical tumor models; (III) *in vitro* or ex vivo functional assays; (IV) descriptive or bioinformatic analyses. When multiple studies investigated the same chemokine-Treg axis within a given tumor type, only the highest level of evidence was retained. Using this approach, eight core chemokine-Treg axes were identified across diverse tumor types and summarized with their corresponding tumor contexts, providing a structured framework to compare shared and tumor-specific mechanisms of Treg recruitment and immunosuppression (**Figure 2**). The axes include: CCL17/22-CCR4 (39, 58–63), CCL8-CCR5 (64), CCL20-CCR6 (40, 65–67), CCL1-CCR8 (68–71), CCL28-CCR10 (72), CXCL1-CXCR2 (73), CXCL4/CXCL11-CXCR3 (74, 75), and CXCL12-CXCR4 (41, 76, 77).

#### 4.2.1 Breast cancer

Breast cancer is characterized by multiple chemokine-driven mechanisms that promote regulatory T cell recruitment and the establishment of an immunosuppressive tumor microenvironment. Elevated expression of CCL17 and CCL22 in primary breast tumors drives the accumulation of CCR4^+^ Tregs and is consistently associated with increased Treg infiltration and unfavorable clinical outcomes (39, 62, 77). In addition, invasive breast cancer tissues exhibit increased CCL1 expression, which selectively attracts CCR8^+^ FoxP3^+^ Tregs; high CCL1 levels correlate with reduced patient survival, suggesting enrichment of highly suppressive Treg subsets (68).

Chemokine-mediated Treg recruitment also contributes to metastatic progression. In preclinical models of breast cancer lung metastasis, the CCR5-CCL8 axis is associated with site-specific accumulation of Tregs within the lung microenvironment. Pharmacological inhibition of CCR5 reduces Treg infiltration at metastatic sites and enhances antitumor responses, indicating distinct chemokine-Treg programs operating in primary and metastatic settings (64).

#### 4.2.2 Digestive tract tumors

Digestive tract tumors, including gastric and colorectal cancers, commonly display chemokine-driven regulatory T cell accumulation. In gastric cancer, activation of PPARδ induces CCL20 expression, promoting recruitment of CCR6^+^ Tregs and formation of an immunosuppressive niche associated with tumor progression and poor prognosis (40). In colorectal cancer, chemokine signaling also affects Treg function. Tumor-derived CXCL4 suppresses cytotoxic T lymphocyte activity and enhances Treg proliferation and TGF-β production via CXCR3-dependent mechanisms, thereby reinforcing local immune suppression and facilitating tumor growth (75).

#### 4.2.3 Gynecological malignancies

In gynecological cancers, particularly ovarian cancer, regulatory T cell infiltration is shaped by both inflammatory and hypoxia-responsive chemokine pathways. Ovarian tumor cells and tumor-associated macrophages produce CCL22, promoting accumulation of CCR4^+^ Tregs within the tumor microenvironment, whereas tumors with low CCL22 expression show limited Treg infiltration (9). In parallel, intratumoral hypoxia induces CCL28 expression and activates the CCR10-CCL28 axis. Recruitment of CCR10^+^ Tregs through this pathway is associated with enhanced immune tolerance and increased angiogenesis, contributing to tumor progression (72).

#### 4.2.4 Lung cancer and pleural metastatic niches

In lung cancer-associated microenvironments, chemokine-Treg interactions are particularly evident within localized tumor niches. In non-small cell lung cancer with malignant pleural effusion, elevated CXCL1 expression correlates with increased Treg infiltration and reduced patient survival. Mechanistically, downregulation of miR-141 activates the CXCL1-CXCR2 axis, enhancing Treg recruitment and contributing to immune evasion and metastatic progression (73).

#### 4.2.5 Virus-associated tumors

Virus-associated malignancies show pronounced chemokine-driven regulatory T cell recruitment. In Epstein-Barr virus-associated nasopharyngeal carcinoma, viral infection activates a c-JUN-miR-200a-CXCL12-c-JUN positive feedback loop, resulting in sustained CXCL12 expression and recruitment of CXCR4^+^ Tregs, thereby reinforcing an immunosuppressive tumor microenvironment (41). Similarly, in hepatitis B virus-related hepatocellular carcinoma, chronic inflammatory signaling enhances *TGF-β*-mediated suppression of miR-34a, leading to upregulation of CCL22 and CCR4-dependent Treg infiltration. This process promotes immune tolerance and facilitates tumor progression and portal vein metastasis (61).

## 5 Therapeutic targeting of chemokine-Treg axes in cancer

Chemokine-driven Treg recruitment, including CCR4-CCL17/22, CCR6-CCL20, CXCR4-CXCL12, and CCR8-CCL1, contributes to tumor immunosuppression. Clinical targeting is limited by Treg heterogeneity and pathway redundancy, highlighting the need for precision strategies. Future approaches should prioritize tumor-enriched receptors such as CCR8, address pathway redundancy through multi-receptor or upstream targeting, and explore rational combination therapies, as demonstrated by the synergy between CCR8 blockade and anti-PD-1 immunotherapy (78). Biomarker-guided patient stratification that incorporates receptor expression, chemokine signatures, and spatial localization will be essential to optimize therapeutic efficacy. Advanced technologies, including single-cell analysis and spatial transcriptomics, can map chemokine gradients and immune cell localization, revealing context-specific mechanisms and therapeutic vulnerabilities (79). Integrating specific targeting, combination therapy design, and biomarker-guided strategies offers the potential to dismantle the immunosuppressive niche and improve outcomes in resistant malignancies.

## 6 Summary and future perspectives

This review synthesizes eight experimentally validated chemokine-Treg axes identified between 2005 and 2025, detailing the tumor types in which each axis has been confirmed. Two illustrative figures complement this synthesis: one visualizes the mechanisms by which chemokines recruit Tregs and facilitate tumor immune evasion, while the other maps all eight axes across different cancers, offering a comprehensive overview of their functional landscape.

Importantly, this integrative framework serves as a practical guide for future research. By focusing on these eight core axes, investigators can efficiently screen for tumor-specific pathways responsible for Treg recruitment and immune suppression, significantly narrowing the scope of exploratory studies. These chemokine-Treg axes not only provide mechanistic insights into tumor immune evasion but also highlight potential therapeutic targets and predictive biomarkers. As additional experimental data emerge, this model can be refined, supporting both mechanistic research and the rational development of strategies to overcome Treg-mediated immunosuppression in cancer therapy.

## Glossary

Tregs: Regulatory T cells
TME: Tumor microenvironment
CCL: C-C Motif Chemokine Ligand
CCR: C-C Motif Chemokine Receptor
MDSCs: Myeloid-Derived Suppressor Cells
TAMs: Tumor-Associated Macrophages
*GM-CSF*: Granulocyte-Macrophage Colony-Stimulating Factor
*TGF-β*: *Transforming Growth Factor-beta*
*IL-10*: *Interleukin-10*
*FoxP3*: *Forkhead Box Protein P3*
NKs: Natural Killer Cells
*PD-1*: *Programmed Cell Death Protein 1*
*PD-L1*: *Programmed Death-Ligand 1*
*CTLA-4*: *Cytotoxic T-Lymphocyte-Associated Protein 4*
ECM: Extracellular matrix
ROS: Reactive Oxygen Species
nTregs: Natural Tregs
iTregs: Inducible Tregs
*Bcl-2*: B-cell lymphoma 2
*GITR*: Glucocorticoid-Induced TNFR-Related protein
XCL: X-C Motif Chemokine Ligand
CXCL: C-X-C Motif Chemokine Ligand
XCR: X-C Motif Chemokine Receptor
CXCR: C-X-C Motif Chemokine Receptor
CTLs: Cytotoxic T Lymphocytes
PI3K: Phosphatidylinositol 3-Kinase
AKT: AKT serine/threonine kinase
JAK: Janus Kinase
STAT: Signal Transducer and Activator of Transcription
MAPK: Mitogen-Activated Protein Kinase
ERK: Extracellular Signal-Regulated Kinase
*PPARδ*: Peroxisome Proliferator-Activated Receptor Delta
NSCLC: Non-Small Cell Lung Cancer
MPE: Malignant Pleural Effusion
EBV: Epstein-Barr Virus
HBV: Hepatitis B Virus
HCC: Hepatocellular Carcinoma
HNSCC: Head and Neck Squamous Cell Carcinoma
UTR: Untranslated Region
